# Supplementary material for: Maintenance of cell type-specific connectivity and circuit function requires Tao kinase
Source: Nat Commun. 2019 Aug 5;10:3506. doi: 10.1038/s41467-019-11408-1 (PMC6683158; doi:10.1038/s41467-019-11408-1)
Supplement: Supplementary file 1 — Supplementary Information [file 41467_2019_11408_MOESM1_ESM.pdf]

Supplementary Information

Maintenance of cell type-specific connectivity and circuit function requires Tao kinase

Tenedini et al.

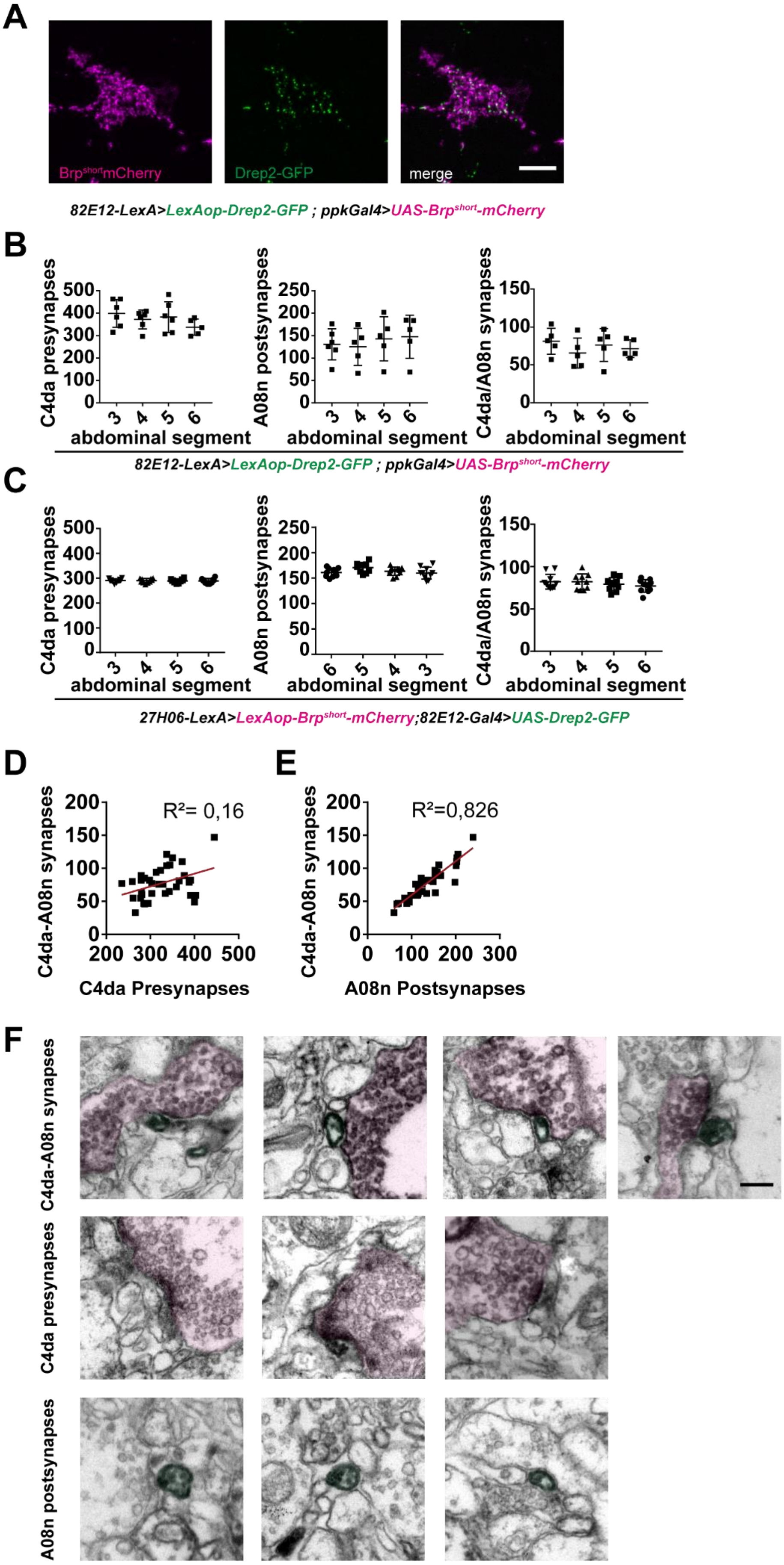

A
BrpshortmCherry
Drep2-GFP
merge
82E12-LexA>LexAop-Drep2-GFP ; ppkGal4>UAS-Brpshort-mCherry
B
C4da presynapses
A08n postsynapses
C4da/A08n synapses
abdominal segment
82E12-LexA>LexAop-Drep2-GFP ; ppkGal4>UAS-Brpshort-mCherry
C
C4da presynapses
A08n postsynapses
C4da/A08n synapses
abdominal segment
27H06-LexA>LexAop-Brpshort-mCherry;82E12-Gal4>UAS-Drep2-GFP
D
C4da-A08n synapses
R²= 0,16
C4da Presynapses
E
C4da-A08n synapses
R²=0,826
A08n Postsynapses
F
C4da-A08n synapses
C4da presynapses
A08n postsynapses

Supplementary Figure 1

(A) Larval VNC hemisegment (96h AEL) with expression of Brp^{short}-mCherry in C4da (magenta), Drep2-GFP in A08n (green) and merge (*82E12-LexA>LexAop-Drep2-GFP; ppk-Gal4>UAS-Brp*^{short}*-mCherry*). Scale bar = 5 µm. (B) Quantification of C4da presynapses, A08n postsynapses and C4da-A08n synpases in abdominal segments 3-6 using *82E12-LexA>LexAop-Drep2-GFP; ppk-Gal4>UAS-Brp*^{short}*-mCherry*. n = 6. (C) Quantification of C4da presynapses, A08n postsynapses and C4da-A08n synpases in abdominal segments 3-6 using *27H06-LexA>LexAop-Brp*^{short}*-mCherry ; 82E12-Gal4>UAS-Drep2-GFP*. n = 10. (D) Correlation between C4da-A08n synapses and C4da presynapses with linear regression. R^2=0.16, n = 40 hemisegments from 10 animals (E) Correlation between C4da-A08n synapses (colocalization) plotted against A08n postsynapses with linear regression. R^2=0.826, n = 40 hemisegments from 10 animals. (F) EM images showing DAB-labelled C4da presynapses (colored in magenta) connected to A08n postsynaptic compartments (colored in green), and C4da or A08n synaptic compartments without labelled counterparts. Scale bar = 200 nm.

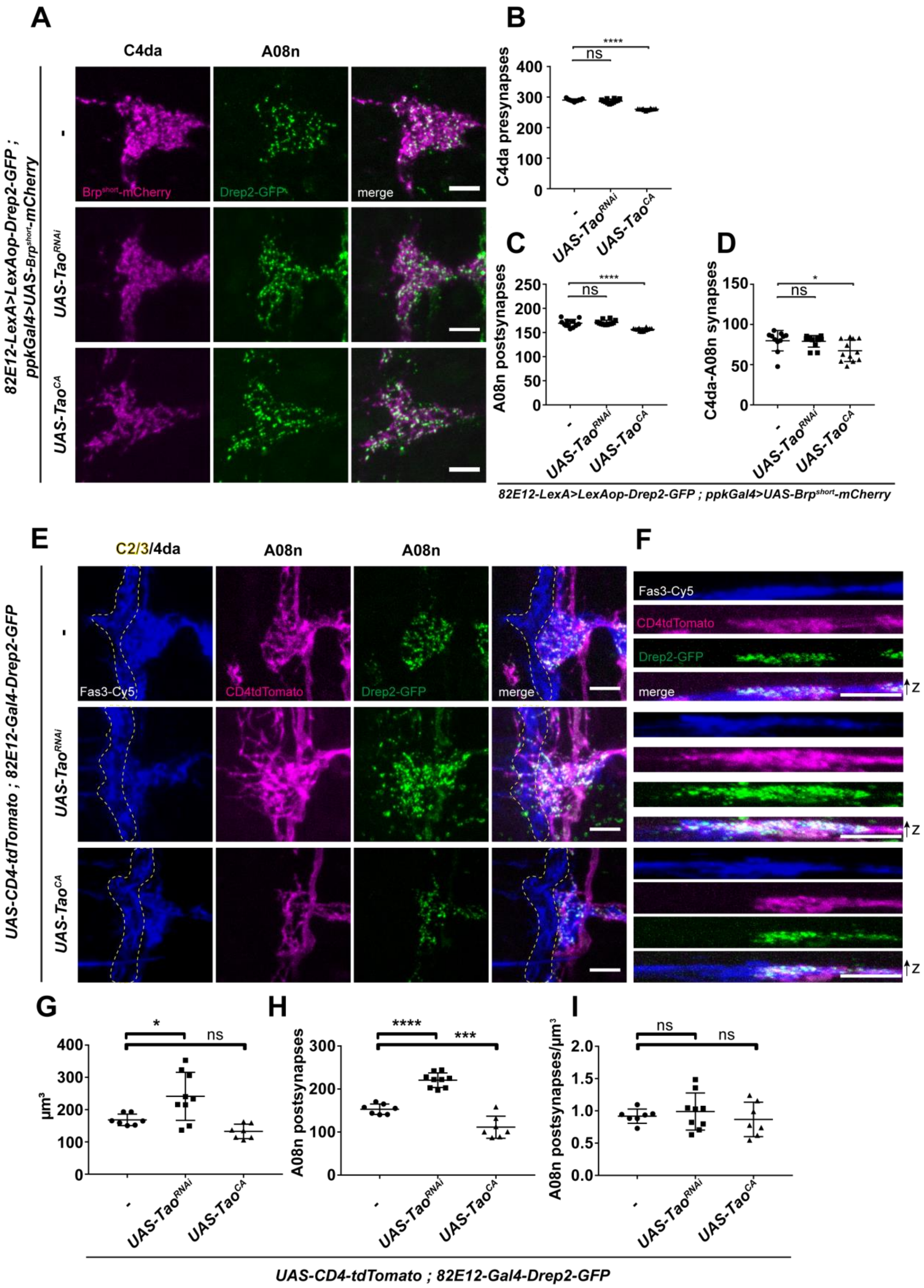

Supplementary Figure 2

(A) Confocal images of larval VNC hemisegments (96h AEL) in *control,* or with *Tao*^{RNAi} and Tao^{CA} expression in C4da neurons using synaptic markers labeling C4da presynapses (magenta) and A08n postsynapses (green). Scalebar = 5 µm. (B-D)

Quantification of (B) C4da pre-, (C) A08n post- and (D) colocalized C4da-A08n synaptic markers in control or with *Tao*^{RNAi} and Tao^{CA} expression in C4da neurons. **P*<0.05, **** *P* < 0.0001. ±SD, ANOVA with multiple comparisons and Dunnett's *post-hoc* test (for exact *P* values and statistics see Supplemental Table 1). Control n = 11, *UAS-Tao*^{RNAi} n = 12, *UAS-Tao*^{CA} n = 12. (E) Confocal images of hemisegments in control or with *Tao*^{RNAi} and Tao^{CA} expression in A08n neurons. A08n neurons were labeled using the morphological marker *CD4-tdTomato* (magenta) and postsynaptic marker *Drep2-GFP* (green), together with anti-Fas3 immunostaining labeling C2da, C3da and C4da sensory axons (blue). C2da/C3da domain is indicated by yellow dotted line. Scale bar = 5 µm. (F) XZ projections of each channel in (E) are shown. Scale bar = 5 µm. (G-I) Quantification of (G) dendrite volume, (H) A08n postsynapses and (I) A08n postsynapse/volume ratio per hemisegment in control or with *Tao*^{RNAi} and Tao^{CA} expression in A08n neurons. **P*<0.05, ***P*<0.005, **** *P* < 0.0001. ±SD, ANOVA with multiple comparisons and Dunnett's *post-hoc* test (for exact *P* values and statistics see Supplemental Table 1). Control n = 7, *UAS-Tao*^{RNAi} n = 9, *UAS-Tao*^{CA} n = 7.

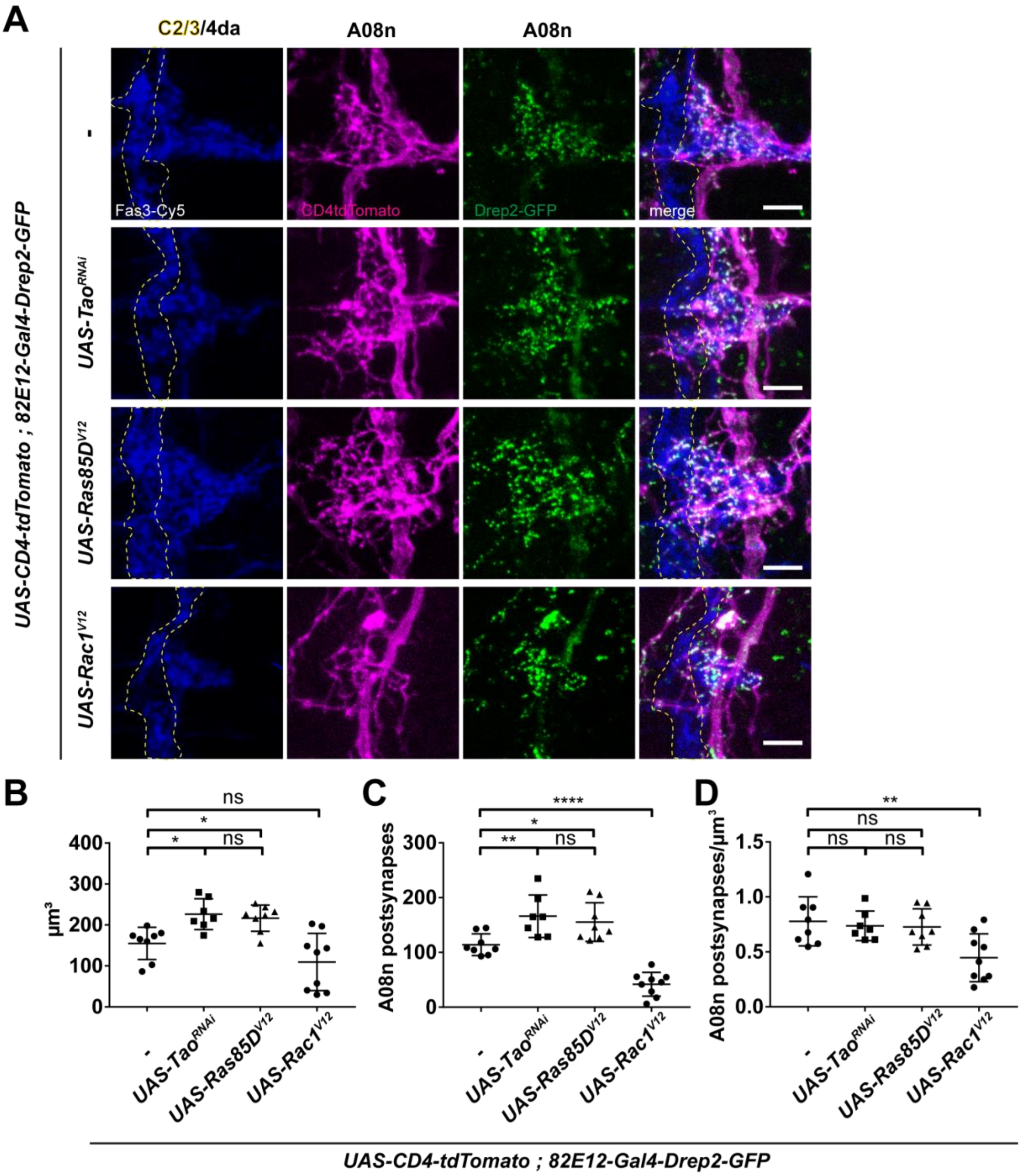

Supplementary Figure 3.

Ras1 and Rac1 overexpression differentially regulate dendrite growth and synaptogenesis of A08n neurons. (A) Confocal images of larval VNC hemisegments (96h AEL) in control or with *Tao*^{RNAi}, Ras85D^{V12} and Rac1^{V12} expression in A08n neurons. A08n neurons were labeled using the morphological marker *CD4-tdTomato* (magenta) and postsynaptic marker *Drep2-GFP* (green), together with anti-Fas3 immunostaining labeling C2da, C3da and C4da sensory axons (blue). C2da/C3da domain is indicated by yellow dotted line. Scale bar = 5 µm. (B-D) Quantification of (B) dendrite volume, (C) A08n postsynapses and (D) postsynapse/volume ratio per

hemisegment in control or with *Tao*^{RNAi}, Ras85D^{V12} and Rac1^{V12} expression in A08n neurons. * $P<0.05$, ** $P<0.01$, **** $P < 0.0001$. $\pm$SD, ANOVA with multiple comparisons and Dunnett's *post-hoc* test (for exact P values and statistics see Supplemental Table 1). control n = 8, *UAS-Tao*^{RNAi} n = 7, *UAS-Ras85D*^{V12} n = 8, *UAS-Rac1*^{V12} n = 9.

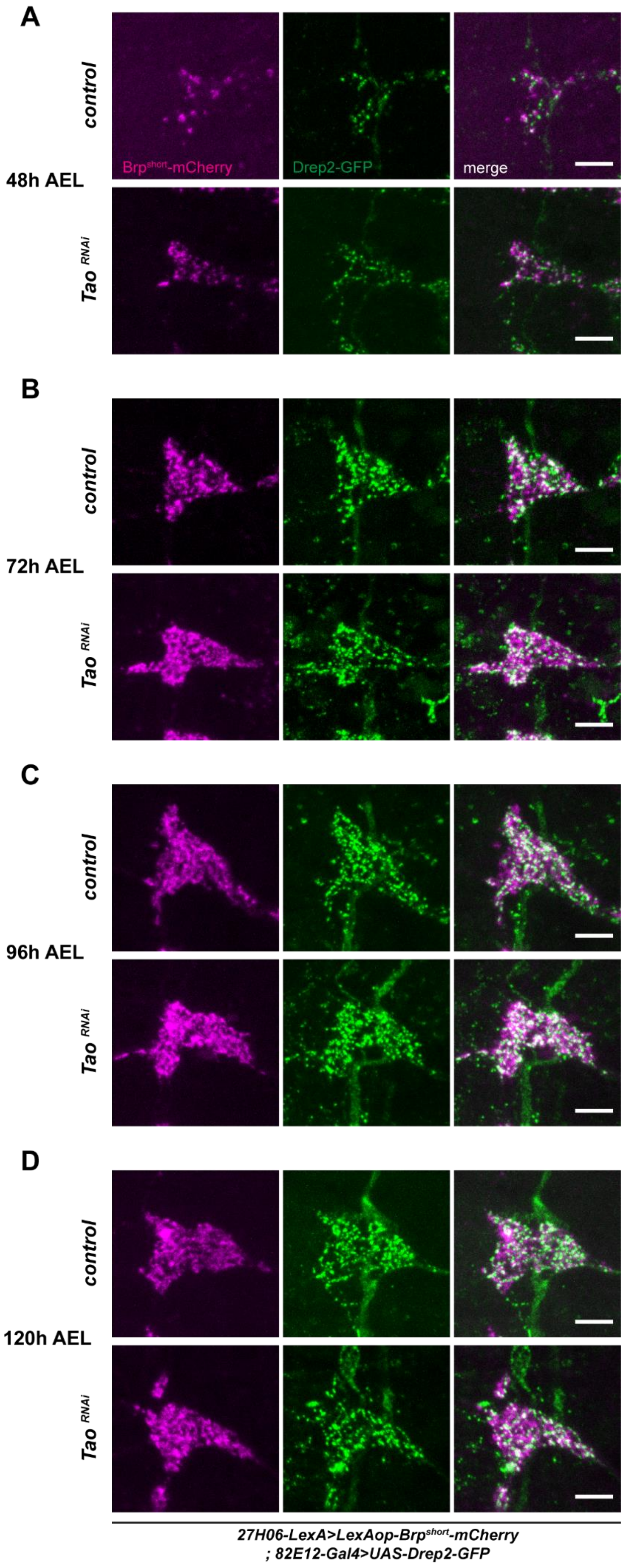
A
control
Brp^short-mCherry
Drep2-GFP
merge
48h AEL
Tao RNAi
B
control
72h AEL
Tao RNAi
C
control
96h AEL
Tao RNAi
D
control
120h AEL
Tao RNAi
27H06-LexA>LexAop-Brp^short-mCherry
; 82E12-Gal4>UAS-Drep2-GFP

Supplementary Figure 4

Confocal images of representative larval hemisegments during development from 48 AEL until 120h AEL in control or with *Tao*^{RNAi} expression in A08n neurons. Synaptic markers labeling C4da presynapses (magenta), A08n postsynapses (green) are shown at (A) 48h, (B) 72h, (C) 96h and (D) 120h AEL. Scale bar = 5 µm.

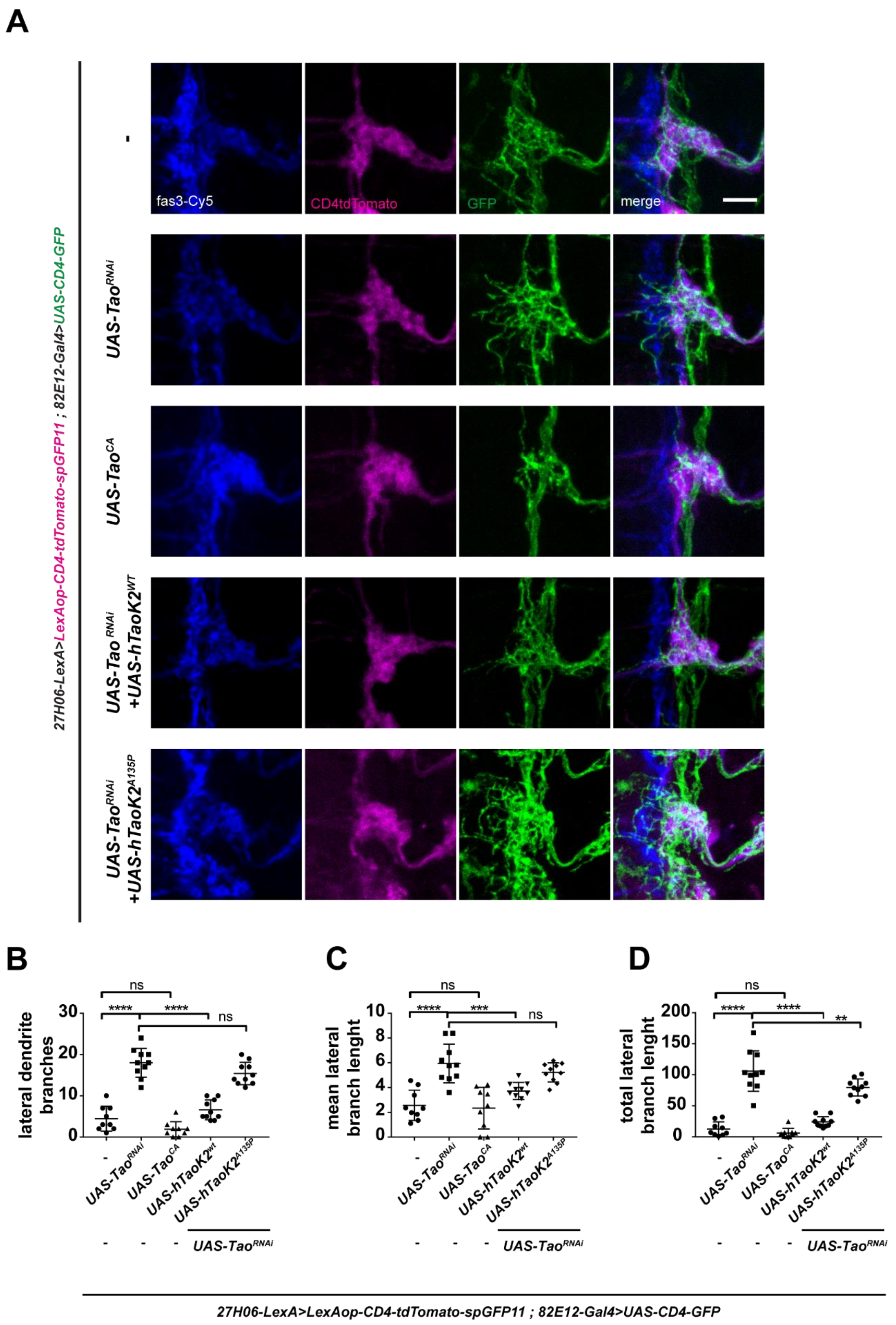

Supplementary Figure 5

(A) Confocal Images of larval VNC hemisegments (96h AEL) in control, with *Tao*^{RNAi} and Tao^{CA}, or co-expression of *Tao*^{RNAi} with hTaoK2^{wt} or hTaoK2^{A135P} in A08n neurons. Images show anti-Fas3 immunostaining labeling C2da, C3da and C4da

sensory axons (blue), with anatomical marker expression to label C4da axons (CD4-tdTomato, magenta) and A08n neurons (CD4-tdGFP, green) . Scale bar = 5 µm. (B-D) Quantification of A08n neuron ectopic dendritic branches per hemisegment laterally displaced exiting the C4da domain (lateral dendrite branches). (B) Lateral dendrite numbers, (C) mean branch length and (D) total lateral branch length in control, with $\mathit{Tao}^{\mathit{RNAi}}$ and $\mathrm{Tao}^{\mathrm{CA}}$, or co-expression of $\mathit{Tao}^{\mathit{RNAi}}$ with $\mathrm{hTaoK2}^{\mathrm{wt}}$ or $\mathrm{hTaoK2}^{\mathrm{A135P}}$ in A08n neurons. Control: n = 9, *UAS-Tao*^{RNAi}: n = 10, *UAS-Tao* ^{CA}: n = 9, *UAS-Tao*^{RNAi} + *UAS-hTaoK2* ^{wt}: n = 10, *UAS-Tao*^{RNAi} + *UAS-hTaoK2* $^{\mathit{A135P}}$ n = 10. ***P<0.001, **** P < 0.0001. ±SD, ANOVA with multiple comparisons and Dunnett's *post-hoc* test (for exact *P* values and statistics see Supplemental Table 1).

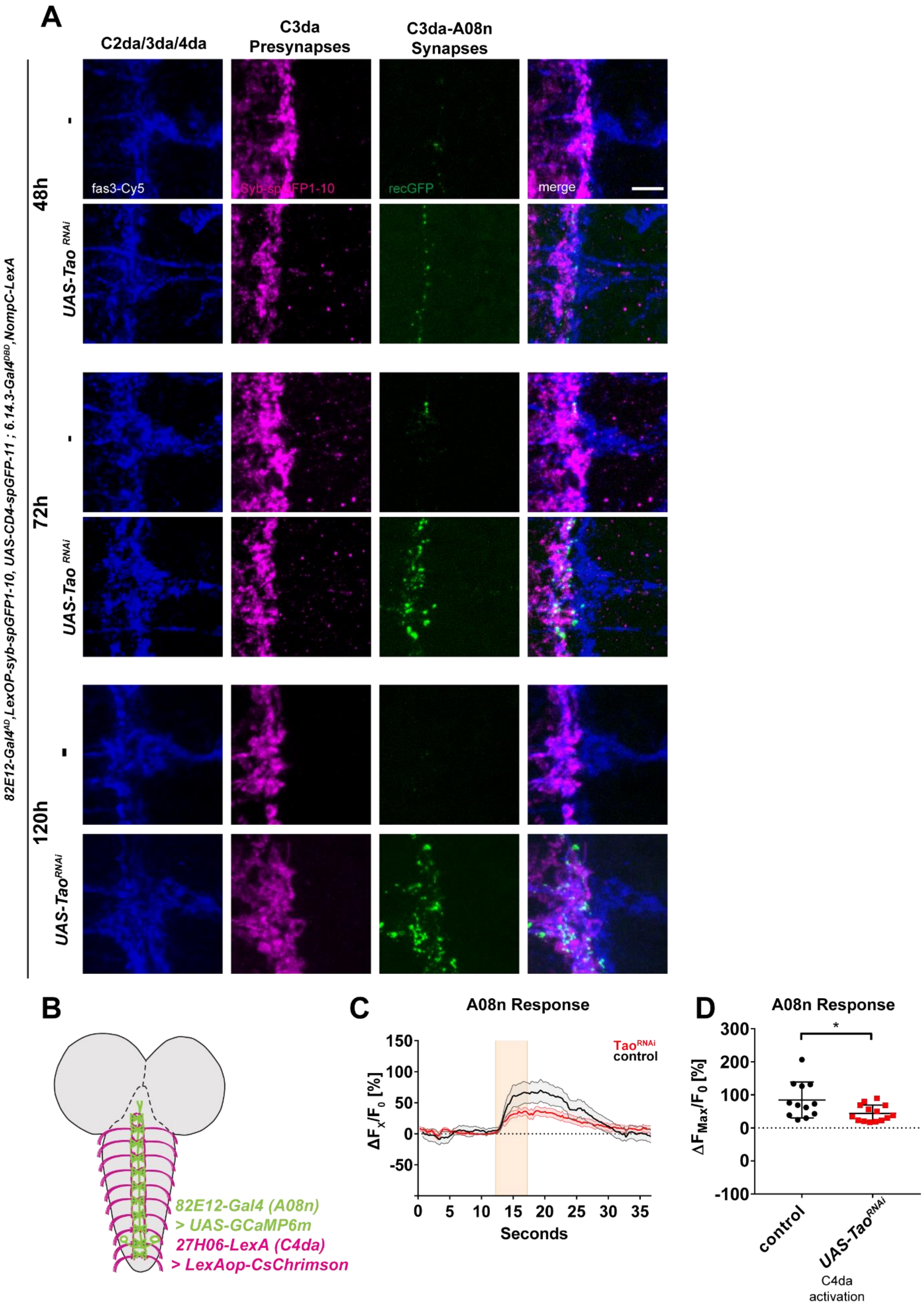

Supplementary Figure 6

(A) Confocal images of Syb-GRASP-labeled C3da-A08n synapses (48, 72 and 120h AEL). Representative images of larval VNC hemisegments in control or with *Tao*^{RNAi}

expression in A08n neurons showing anti-Fas3 labeling of C2da, C3da and C4da sensory axons (blue), presynaptic Syb-spGFP1-10 expressed in C3da (magenta) and reconstituted native GFP signal marking C3da-A08n Synapses (green). Scale bar = 5 µm. (B) Schematic larval brain showing A08n neurons (green) and C4da sensory dendrite VNC projections (magenta) and indicating expression of *UAS-GCamp6m* in A08n and *LexAop-CsCrimson* in C4da neurons. (C) Calcium responses of Gcamp6m-expressing A08n neurons after optogenetic activation of C4da neurons using CsChrimson (5s, 630 nm, indicated by shaded area), with or without Tao^{RNAi} expression in A08n neurons. Data show mean change in percent [($\Delta F/F_0$)-1, (±SEM indicated by shaded regions]. *Control* n = 11, $UAS\text{-}Tao^{RNAi}$ n = 9. (D) Quantification of maximum A08n responses to C4da activation in percent [($\Delta F_{Max}/F_0$)-1)] comparing control and Tao^{RNAi} expression in A08n neurons. Control n=11, $UAS\text{-}Tao^{RNAi}$ n=9. *P*= 0,0199. ±SD. Unpaired two-tailed *t*-test.

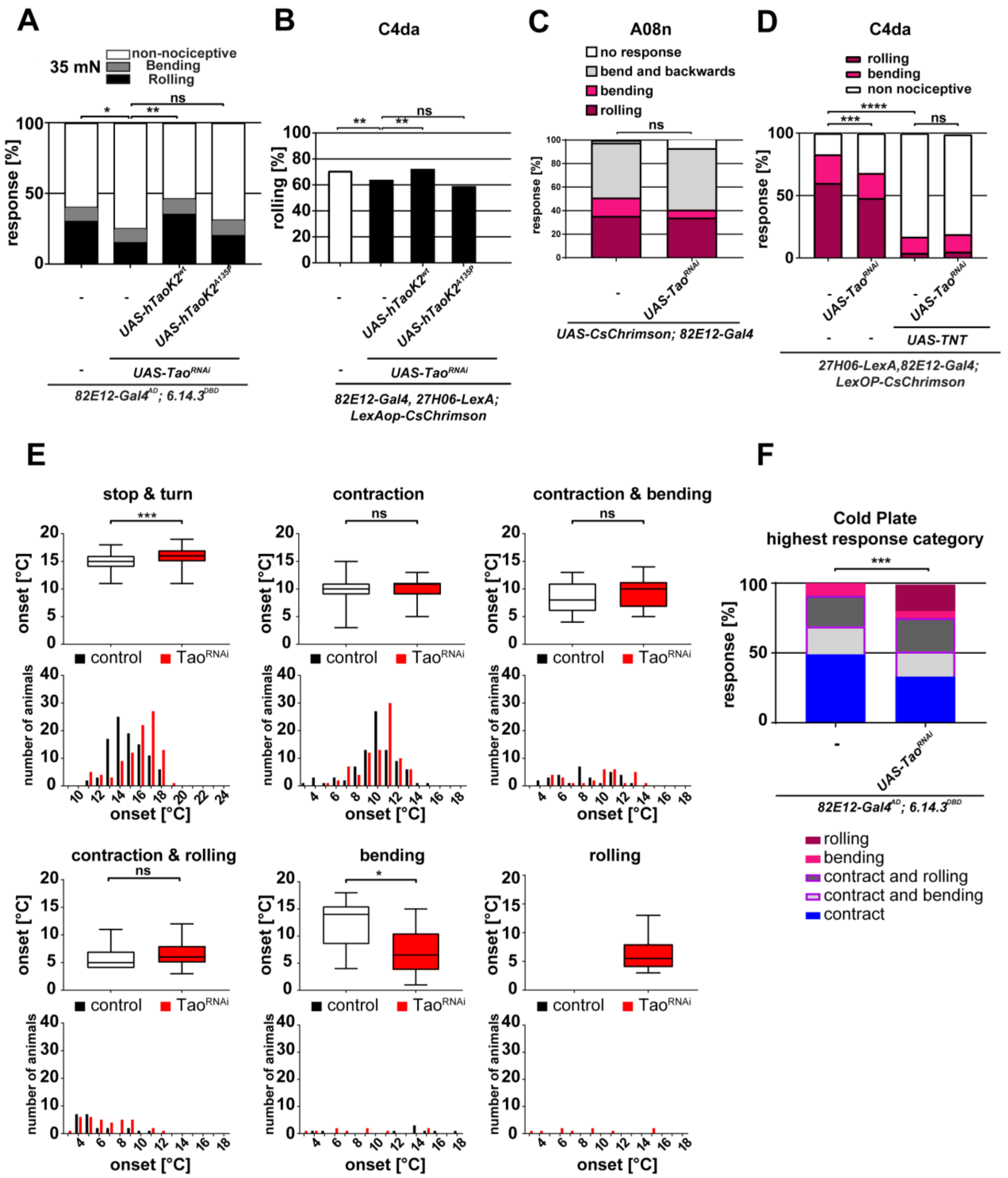

Supplementary Figure 7

(A) Mechanonociceptive behavioral response of third instar larvae (96h AEL) in control or with *Tao*^{RNAi} without or with co-expression of hTaoK2^{wt} or hTaoK2^{A135P} in A08n neurons. Responses to the second mechanical stimulation with a 35 mN *von Frey* filament are shown (Nociceptive rolling and bending or non-nociceptive responses). *P < 0.05, ***P < 0.001. *Control* n = 100, *UAS-Tao*^{RNAi} n = 98, *UAS-Tao*^{RNAi} + *UAS-hTaoK2*^{wt} n = 99, *UAS-Tao*^{RNAi} + *UAS-hTaoK2*^{A135P} n = 99. Control vs

UAS-Tao^{RNAi}: *P* = 0.0439, *UAS-Tao^{RNAi}* vs *UAS-Tao^{RNAi}* + *UAS-hTaok2*^{wt}: *P* = 0.0037, *UAS-Tao^{RNAi}* vs *UAS-Tao^{RNAi}* + *UAS-hTaok2^{A135P}*: *P* = 0.6268, X^2 test.(B) Behavioral responses of third instar larvae (96h AEL) to optogenetic activation of C4da neurons expressing *CsChrimson* (5 s, 625 nm light pulse) in control, or *Tao^{RNAi}* without or with co-expression of hTaoK2^{wt} or hTaoK2^{A135P} in A08n neurons. **$P < 0.005$. *Control*: n = 95, *UAS-Tao^{RNAi}*: n = 91, *UAS-Tao^{RNAi}* + *UAS-hTaoK2^{wt}*: n = 80, *UAS-Tao^{RNAi}* + *UAS-hTaoK2^{A135P}*: n = 98. *Control vs UAS-Tao^{RNAi}*: *P* = 0,0049, *UAS-Tao^{RNAi}* vs *UAS-Tao^{RNAi}* + *UAS-hTaok2*^{wt}: *P* = 0.0016, *UAS-Tao^{RNAi}* vs *UAS-Tao^{RNAi}* + *UAS-hTaok2^{A135P}*: *P* = 0.2667, X^2 test (C) Behavioral responses of third instar larvae (96h AEL) to optogenetic activation of A08n neurons expressing CsChrimson (5 s, 625 nm light pulse) in control or *Tao^{RNAi}* expression in A08n neurons. *Control*: n = 95, *UAS-Tao^{RNAi}*: n = 91 *P* = 0.1005, X^2 test. (D) Behavioral responses of third instar larvae (96h AEL) to optogenetic activation of C4da neurons expressing CsChrimson (5 s, 625 nm light pulse) in control, or with *Tao^{RNAi}* and TNT expression in A08n neurons. **$P < 0.005$, ****$P < 0.0001$. *Control* n = 206, *UAS-Tao^{RNAi}* n = 185, *UAS-TNTe* n = 288, *UAS-Tao^{RNAi}* + *UAS-TNTe* n = 102. *Control* vs *UAS-Tao^{RNAi}*: *P* = 0.0005, *Control* vs *UAS-TNTe P* = >0.0001, *UAS-TNTe* vs *UAS-Tao^{RNAi}* + *UAS-TNTe P* = 0.7307, X^2 test. (E) Temperature-dependent onset and animal distribution of the observed behaviors: stop & turn, contraction, contraction & bending, contraction & rolling, bending and rolling. Stop & turn: control n = 98, *UAS-Tao^{RNAi}* n = 96. Contract: *control* n = 87, *UAS-Tao^{RNAi}* n = 84. Contract & bending *control* n = 31, *UAS-Tao^{RNAi}* n = 30. Contraction & rolling: *control* n = 22, *UAS-Tao^{RNAi}* n = 35. Bending: *control* n = 9, *UAS-Tao^{RNAi}* n = 11. Rolling: *control* n = 0, *UAS-Tao^{RNAi}* n = 20 **P* = >0.05 ****P* = >0.001 *****P* = >0.0001. ±SD Unpaired two-tailed *t*-test. Box and whisker plots depict median, 25th and 75th percentile (lower and upper end of box, respectively), and 5th and 95th percentile (lower and upper whiskers, respectively) of the data points. (F) Percentage of animals shown in Fig. 7D displaying behavior with the highest response category (rolling>bending>contract+rolling>contract+bending >contract). ***$P = 0.0001$, X^2 test.
