# Supplementary material for: Maintenance of cell type-specific connectivity and circuit function requires Tao kinase
Source: Nat Commun. 2019 Aug 5;10:3506. doi: 10.1038/s41467-019-11408-1 (PMC6683158; doi:10.1038/s41467-019-11408-1)
Supplement: Supplementary file 4 — Description of Additional Supplementary Files [file 41467_2019_11408_MOESM4_ESM.docx]

**Description of Additional Supplementary Files**

File Name: Supplementary Data 1
Description: Statistical analysis and P values of all data provided in Figures 1-7 and Supplementary Figures 1-7
