# Supplementary material for: Maintenance of cell type-specific connectivity and circuit function requires Tao kinase
Source: Nat Commun. 2019 Aug 5;10:3506. doi: 10.1038/s41467-019-11408-1 (PMC6683158; doi:10.1038/s41467-019-11408-1)
Supplement: Supplementary file 2 — Peer Review [file 41467_2019_11408_MOESM2_ESM.pdf]

Reviewers' comments:

Reviewer #1 (Remarks to the Author):

This is an interesting paper on the effects of Tao kinase manipulation on synaptic connectivity between sensory neurons and interneurons. The paper could be published in its present form, but Fig. 7 is confusing, as detailed below, and the paper would be improved if Figs. 7D-F were removed.

Specific comments:

The last section of Results is difficult to read. Fig. 7E should be deleted, as it is confusing and there are really no changes worth noting (one * for Tao RNAi, but these effects don't look convincing).

I don’t understand the circuit diagram. I don’t know why the inhibition circuits are drawn in the way they are. The changes in proposed inhibition due to Tao RNAi don’t seem justified by the data. This should probably be taken out.

The Fig. 6 data clearly shows that Tao RNAi causes activation of A08n neurons in response to C3da input due to the ectopic synapses. But I don’t understand Fig. 7D, which appears to be part of the evidence they are using to draw the diagram. Expressing TNT in A08n neurons eliminates mixed behavior. This implies that the mixed behaviors are due to contributions of A08n neurons (maybe they are related to nociceptive behaviors, which are normally produced via these neurons?). But, is this constitutive activity of A08n neurons? My understanding from the paper is that normally C3da stimulation has no effect on A08n responses (Fig. 6D-E). So, if TNT in A08n neurons affects behavior driven by C3da input that must be because those neurons are sending signals that modify the behavior, but which are not in response to C3da activation.

Then, the partial inhibition of mixed behavior by Tao RNAi makes no sense, since Tao RNAi should lead to A08n responses to C3da activation, while TNT blocks A08n responses. If the mixed behaviors are due to A08n then they should increase in response to C3da stimulation in the Tao RNAi condition. Perhaps Fig. 7D should be deleted as well as Fig. 7E. Alternatively, maybe I just don’t understand their reasoning and this could be clarified by changing the text.

Reviewer #2 (Remarks to the Author):

Tenedini et al.
“Maintenance of cell type-specific connectivity and circuit function requires Tao kinase”
Manuscript Review
Nature Communications

To maintain the inextricable link between organismal growth and neuronal scaling and meet the ever-changing demand of a developing nervous system, synaptic organization and circuit specificity must be maintained throughout the life of an organism. To date, though, the mechanisms that support this growth and maintenance remain unclear. In this manuscript, Tenedini and colleagues fulfill multiple goals on the way to more information. First, they offer evidence of scaling in a novel system, the Drosophila larval nociceptive network. Second, they establish a role for the Tao kinase in adjusting dendritic growth, and with it, the synaptic network connecting C4da presynaptic neurons to A08n postsynaptic neurons. Finally, they posit circuit hypotheses due to the functional adjustments that occur following synaptic disruption (via Tao) in different neurons of the nociceptive circuit.

This manuscript is excellent, addressing an important question and obtaining interesting findings via well conducted and well-controlled studies. I must especially congratulate the authors on their controls and experiments with Brp-Short, GRASP, and immuno-EM to establish the validity and comparability of the synaptic techniques. These will make an excellent addition to the field and will hopefully finally dispel doubt as to the veracity of the Brp-Short label. I think this paper would be more than suitable for publication following a few simple considerations.

Concerns:

1) What is the nature of the Tao kinase perturbation phenotype in A08n? The data seems to suggest that it's a question of adding more dendritic space adds more synapses, and that would be in keeping with the authors' model. But is it this simple? Is Tao solely focusing on the dendritic size and not directly acting on some pathway to add synapses? Does the size of C4da neurons change when Tao is cell autonomously manipulated (though via only one manipulation)? Also, regarding C4da, can the authors speculate on why C4da and A08n are differentially affected by Tao manipulation?

2) Similarly as above, via Figure 4, the authors indicate that Tao is required during development to restrict A08n postsynaptic numbers. Does this happen by actively restricting the synapse number or simply by restricting growth? As in, is this a general mechanism, or a facet specific to the Tao-related mechanism? Either one is interesting, but it would be nice to have some data or speculation at least, which is happening. Are there any ways to increase dendritic size that don't increase synapse number?

3) It's unclear throughout (to non-aficionados) what else C4da connects to – could the authors make a discussion of this more prominent to understand the circuit a bit better?

4) Figure 4 (and graphs like it) are very hard to read. The authors should adjust the formatting to promote comprehension.

Reviewer #3 (Remarks to the Author):

In this study the authors interrogate molecular mechanisms that underlie synaptic scaling during development using a somatosensory circuit in Drosophila larvae. The authors first established light-microscopic methods for quantifying the synapses between larval nociceptors and one of its important second-order neurons, called A08n. They then described how synapse numbers change in larval development. They identify the kinase Tao as a negative regulator of A08n post-synaptic formation during developmental scaling, and demonstrate that the human homolog, but not its kinase-impaired autism-linked variant, rescues the defects caused by loss of Tao. They further show that loss of Tao activity results in circuit aberrations and behavioral changes.
These findings have significant implications for understanding the mechanisms of synaptic scaling during development. The results that show the functional conservation of mammalian and Drosophila Tao Kinase suggest the findings might be relevant to diseases. Most experiments are well designed (though see below), and the work is well articulated. The authors' savvy use of genetic approaches in interrogating synaptic and circuit functions is a strength of the work. I'd support the publication of this paper in Nature Communications after the authors address the following concerns.

Major concerns:

1) In several places the results from statistical analysis do not support the conclusions. The conclusions should be supported by statistical analysis, or they should be revised or removed.
p.7: "C4da neuron presynaptic puncta plateaued between 96 h and 120 h, while A08n postsynaptic

counts dropped mildly at 120 h", but in Fig.2D there is no significant change between 96 h and 120 h. Also, "C4da presynaptic output to A08n neurons remained steady across larval development", but in Fig.2E there are significant differences among 48 h, 72 h, 96 h and 120 h.
p.7: "In contrast, we observed a significant increase in synapse/postsynapse ratios for A08n neurons between 48 h and 96 h, indicating a gradual increase in…". However, the result indicates an increase from 48 h to 72 h, but did not compare between 48 hr and 96 hr.
p. 10: "density of Drep2-GFP-labeled A08n postsynapses suggesting that Tao activity did not strongly perturb synaptic density (Fig. S2D)". The result indicates significant change with one * in the figure.

2) The authors used t-test throughout Fig.2, but ANOVA followed by Bartlett's post hoc should be used.

3) To this reviewer, the correlation shown in Fig 1H & I are not strong enough to indicate a potential postsynaptic control of synaptic connectivity (p.6), and the stated implications of these results should be revised. The postsynaptic Drep2-GFP signals in A08n are much more punctate than the presynaptic Brp[short]-mCherry signals in C4da. It is thus possible that the quantification of Drep2-GFP was more accurate than Brp[short]. In addition, the method used for quantifying C4da-A08n synapses in these results, which is not described, may affect the result. If the synapses are counted by co-localization of Brp[short]-mCherry and Drep2-GFP, the presynaptic correlation might be weakened if C4da neurons have more post-synaptic partners than A08n have presynaptic partners.

4) The authors concluded that ectopic synapses between C3da and A08n are functional and contribute to nociceptive behaviors. However, the first two bars in Fig.7D show that activation of C3da in larvae with Tao-defective A08n neurons does not increase nociceptive behaviors, including rolling and bending, while it increases the turning associated with C3da, which is opposite to authors' conclusion. Moreover, the first two bars in Fig.7E shows the opposite to the claim that defects in A08n caused by Tao RNAi turns C4da-specific behaviors (nociceptive) towards C3da-specific behaviors (mechanosensory). In control, C3da-specific behaviors dominate nociceptive behaviors while Tao RNAi in A08n neurons increases the rolling behaviors (i.e., towards C4da-specific behaviors).

5) There doesn't seem to be any basis for the inhibition loop from A08n to C3da/Ch projections in the model (Fig.7F). Although this inhibition loop explains why Tao RNAi in A08n increases rolling when C3da and C4da are activated together (Fig.7E), it does not explain why Tao RNAi in A08n decreases the rolling when C3da is activated alone (Fig.7D).

6) In p. 15 and Fig 6 (p. 17), NompC-lexA was used as a C3da driver. However, this driver expresses transgenes in both C3da and chordotonal neurons. The main text and the table for "Drosophila melanogaster stocks" should be revised to reflect this fact. Also, "NompC-lexAop" in the label of Fig 6A should be "NompC-lexA".

Minor concerns:

1) p.5: The labels for Fig 1G-J are rather nondescript. For example, consider changing the y-axis labels for Fig 1H and I from "synapse" to "C4da-A08n synapses" to be clear about the type of synapses that were quantified. Make the same change in the other figures with the same issue (e.g., Fig 3).

2) Indicate the developmental stage of the larvae used in the experiments shown in Fig.3.

3) p. 27: "pUAttB" should probably be "pUASTattB".

4) p. 27: "For activity-dependent GFP reconstitution across synaptic partners…", but this reviewer didn't find the main text or figure legends mention activity-dependent GRASP.

5) It’s not easy to tell what Fig 4G is intended to show. Can the authors label or explain in the legends the anterior-posterior orientation of the images?

6) p.20, bottom 4 lines: “About half of all animals displayed mixed responses composed of successive combinations of bending followed by contraction and rolling. 51% of these larvae sequentially performed all three behaviors, with two thirds contracting before rolling.” To this reviewer, one would expect about 25% larvae exhibiting rolling, based on this description. However, the % rolling shown in Fig 7D are much less than this number. Is the description on p.20 correct?

7) The development of C4da topography was first reported in Yang et al., (2014) Current Biology, 24(9): 1024-1030. That paper describes the presence of topography in early 2nd and early 3rd instar larvae (Fig S1F), which roughly match the 48 and 96 h AEL in this manuscript, and should be cited in discussions about the maintenance of topography during development, for example, in p. 22.

8) p.23: “the larval nociceptive network seems to rely more on functional rather than structural adjustments when challenged by sensory input alterations, although fine-scale topography of C4da neuron axon projections has been shown to require activity-dependent Trim9 function.” Neither this study nor the Kaneko et al. cited in this sentence provides any evidence that supports the lack of activity-dependent adjustment by sensory input alternation. Moreover, the regulation of fine-scale topography of C4da neuron axon projections could be through spontaneous activities rather than sensory inputs.

9) p.7: Typo- “…provide valid representations of C4da-A08n neuron connectivity”

10) p.15: Typo- “…first detectable at 48h AEL. .”

11) Figure 6B: The white circles are difficult to distinguish from the black squares. The groups should be marked more clearly.

12) p. 21: “…contraction responses virtually indistinguishable from activation of C3da neurons alone…”

Point by point response

We would like thank the reviewers for their helpful comments. We have carefully addressed each point and added substantial data to strengthen our conclusions. We have further highlighted all changes in the manuscript.

The major changes in our manuscript include:

1. We have added experimental evidence that Tao kinase likely co-regulates neuronal and synaptic growth, based on comparison with overactivation of Ras-dependent and Rac-dependent growth regulatory pathways. These data are now incorporated in the new Supplementary Figure 3 and have been added to the results and discussion.

2. We have repeated and increased the number of animals analyzed for A08n neuron calcium responses after C4da neuron activation with and without Tao manipulation. Our data now shows a clear functional defect of A08n responses after Tao knockdown suggesting that despite unaltered or slightly increased synapse numbers, their physiological properties are partially impaired. We have added these data in the revised Supplementary Figure 6B-D.

3. Based on the suggestion of reviewer #1 and the concerns of reviewer #3, we have removed Figure 7, panels D-F (and from Supplementary Figure 6 D,E), which contained the original optogenetic behavioral data (for detailed discussion please see the point by point response). Instead, we now provide conclusive data using noxious cold stimulation, which is altered by loss of Tao in A08n neurons. These results are included in the revised Fig. 7D,E and Supplementary Figure 6E.

4. Based on the reviewers' suggestions and our novel data, we have included a new circuit model of how altering A08n neuron connectivity by Tao manipulation affects function and behavior mediated by C3da and C4da neuron-dependent circuits (Fig. 7F).

Please find our responses to the raised points below:

Reviewer #1 (Remarks to the Author):

This is an interesting paper on the effects of Tao kinase manipulation on synaptic connectivity between sensory neurons and interneurons. The paper could be published in its present form, but Fig. 7 is confusing, as detailed below, and the paper would be improved if Figs. 7D-F were removed.

Specific comments:

The last section of Results is difficult to read. Fig. 7E should be deleted, as it is confusing and there are really no changes worth noting (one * for Tao RNAi, but these effects don't look convincing).

I don't understand the circuit diagram. I don't know why the inhibition circuits are drawn in the way they are. The changes in proposed inhibition due to Tao RNAi don't seem justified by the data. This should probably be taken out.

The Fig. 6 data clearly shows that Tao RNAi causes activation of A08n neurons in response to C3da input due to the ectopic synapses. But I don't understand Fig. 7D, which appears to be part of the evidence they are using to draw the diagram. Expressing TNT in A08n neurons eliminates mixed behavior. This implies that the mixed behaviors are due to contributions of A08n neurons (maybe they are related to nociceptive behaviors, which are normally produced via these neurons?). But, is this constitutive activity of A08n neurons? My understanding from the paper is that normally C3da stimulation has no effect on A08n responses (Fig. 6D-E). So, if TNT in A08n neurons affects behavior driven by C3da input that must be because those neurons are sending signals that modify the behavior, but which are not in response to C3da activation. Then, the partial inhibition of mixed behavior by Tao RNAi makes no sense, since Tao RNAi should lead to A08n responses to C3da activation, while TNT blocks A08n responses. If the mixed behaviors are due to A08n then they should increase in response to C3da stimulation in the Tao RNAi condition. Perhaps Fig. 7D should be deleted as well as Fig. 7E. Alternatively, maybe I just don't understand their reasoning and this could be clarified by changing the text.

<u>Response</u>: We thank the reviewer for raising this point and openly discussing his/her thoughts on our data and interpretation. We realize that the interpretation of the optogenetic data is not straight forward and cannot be explained in a simple manner when only considering the C3da-A08n circuit changes induced by loss of Tao in A08n. In our attempt to interpret the data we realized that it might be explained if Tao perturbation in A08n neurons alters inhibitory feedback/feedforward circuits, which likely regulate C3da and C4da neuron dependent behaviors. In proof of their existence, we have performed calcium imaging of A08n neuron responses upon activation of either C4da neurons alone or in combination with C3da (and chordotonal) neurons. Interestingly, A08n responses are suppressed upon co-activation of C3da/cho neurons suggesting that there is indeed inhibitory feedback from the C3da neurons circuit to A08n neurons. This is highly consistent with our and previous observations that optogenetic C3da vs. C3/4da neuron activation results in identical mostly C3da-linked behavioral responses. This suggests that upon A08n neuron manipulation (Tao-RNAi or silencing), this feedback circuit might be perturbed, which alters the behavioral output indirectly. However, we have so far not identified the nature of the relevant feedback neurons, or their precise connectivity and potential functional changes due to Tao perturbation. In light of the complex and difficult to interpret results of our optogenetic behavior, we therefore agree with this referee and have removed panels D-F from Figure 7, including associated Supplementary panels (Supplementary Fig. 6D,E).

Instead, we have now performed a more straight forward analysis of behavioral responses to noxious cold, which depends on C3da/cho neuron function (Turner et al. Curr. Biol. 2016,2018). Possibly due to the more natural type of stimulation compared to optogenetics, we have in this case identified a clear role for Tao-dependent changes in C3da-A08n connectivity and function. Loss of Tao function in A08n neurons resulted in an increase in C4da-specific rolling behavior at the expense of C3da-dependent contraction behavior (see new Fig.7D,E, Supplementary Fig. 7E). Pure noxious cold-induced rolling behavior was never observed in controls, but was significantly increased upon Tao-RNAi manipulation in A08n neurons. Together with our previously shown increase in A08n activation by C3da/cho neurons or by noxious cold under these conditions (now included in Fig. 6C-E and new Fig. 7C), these data strongly argue that aberrant C3da-A08n connectivity is causal for these behavioral changes. While other parameters change as well (e.g. onset of stop and turn behavior, Fig. S7E), these might have to be explained by more complex network alterations, which will require further studies.

Reviewer #2 (Remarks to the Author):

Tenedini et al.
"Maintenance of cell type-specific connectivity and circuit function requires Tao kinase"
Manuscript Review
Nature Communications

To maintain the inextricable link between organismal growth and neuronal scaling and meet the ever-changing demand of a developing nervous system, synaptic organization and circuit specificity must be maintained throughout the life of an organism. To date, though, the mechanisms that support this growth and maintenance remain unclear. In this manuscript, Tenedini and colleagues fulfill multiple goals on the way to more information. First, they offer evidence of scaling in a novel system, the Drosophila larval nociceptive network. Second, they establish a role for the Tao kinase in adjusting dendritic growth, and with it, the synaptic network connecting C4da presynaptic neurons to A08n postsynaptic neurons. Finally, they posit circuit hypotheses due to the functional adjustments that occur following synaptic disruption (via Tao) in different neurons of the nociceptive circuit.
This manuscript is excellent, addressing an important question and obtaining interesting findings via well conducted and well-controlled studies. I must especially congratulate the authors on their controls and experiments with Brp-Short, GRASP, and immuno-EM to establish the validity and comparability of the synaptic techniques. These will make an excellent addition to the field and will hopefully finally dispel doubt as to the veracity of the Brp-Short label. I think this paper would be more than suitable for publication following a few simple considerations.
Response: We thank the reviewer for the highly positive assessment. We fully agree that the Brp-Short label as well as the Syb-GRASP method are excellent and valid tools, at least upon proper validation in the respective system, to investigate cell type-specific connectivity.

Concerns:
1) What is the nature of the Tao kinase perturbation phenotype in A08n? The data seems to suggest that it's a question of adding more dendritic space adds more synapses, and that would be in keeping with the authors' model. But is it this simple? Is Tao solely focusing on the dendritic size and not directly acting on some pathway to add synapses? Does the size of C4da neurons change when Tao is cell autonomously manipulated (though via only one manipulation)? Also, regarding C4da, can the authors speculate on why C4da and A08n are differentially affected by Tao manipulation?
Response: We thank the reviewer for raising these interesting and important points. Previous work in the Drosophila larval system suggested that dendrite placement and growth determines where and how many synapses are made (Couton et al., 2015; Zwart et al., 2013). This argues that as long as competent partner neurons are around, synaptogenesis might follow the direction and degree of dendritic growth. Consistently, our data suggests that at least dendritic volume to synapse ratios are not altered upon Tao manipulation, implying that the increased postsynapse number might be a consequence of exuberant dendritic growth or *vice versa* (see Supplementary Fig. 2). However, it is difficult to distinguish how Tao acts on each specific pathway, as we could not fully uncouple dendrite growth and synapse formation. The only evidence we have relates to the rescue experiment using kinase impaired human Taok2 (A135P), which normalizes overall A08n postsynapse numbers but not

dendritic overgrowth. This suggests that Tao kinase might play a role in both, dendrite growth and synaptogenesis, but regulates the latter in a partially kinase-independent way.

Regarding C4da neuron changes, Tao likely has a similar effect on both, axons and dendrites. We have observed overgrowth of axons in Tao mutant C4da neuron clones but not under RNAi conditions. This is likely due to the nature of RNAi, which takes time and generally does not induce strong axonal phenotypes in C4da neurons. Therefore, we largely focused on the dendritic compartment, which seems to be more susceptible to Tao-RNAi manipulation. Tao overexpression in C4da neurons however does affect the size of the axon terminal and strongly reduces the presynaptic terminal size and synapse numbers. In this case the A08n neurons follow suggesting that C4da axons are required for the spatial confinement of A08n dendrites and thus also their postsynapses. Overall, this indicates anterograde and retrograde signals between the pre- and postsynaptic compartment, which might differentially impinge on Tao activity. While we could speculate on this point we feel that we lack the data to support a clear conclusion and did not further elaborate on this point in our discussion.

We were nonetheless highly interested in addressing how Tao function is linked to dendrite growth and synaptogenesis. We have tried to address this experimentally, which is outlined in our response of the reviewer's related questions below.

2) Similarly as above, via Figure 4, the authors indicate that Tao is required during development to restrict A08n postsynaptic numbers. Does this happen by actively restricting the synapse number or simply by restricting growth? As in, is this a general mechanism, or a facet specific to the Tao-related mechanism? Either one is interesting, but it would be nice to have some data or speculation at least, which is happening. Are there any ways to increase dendritic size that don't increase synapse number?

Response: We thank the reviewer for this comment and we have tried to address this point experimentally using relatively simple perturbations. We have compared the effects on dendrite and postsynaptic growth using the manipulation of growth pathways by expressing constitutively active Ras, which activates canonical MAPK signaling and is implicated in dendrite growth and synaptogenesis (Koh et al., 2002; Kumar et al., 2005). We compared this manipulation to Tao-RNAi and overactivation of Rac1 (using a constitutively active Rac1 transgene), which has been shown to induce excessive dendritic branching in Drosophila sensory neurons and increased synaptic growth at the NMJ (Ball et al., 2010; Lee et al., 2003). Overactivation of Ras displayed increased dendritic and postsynaptic growth in A08n neurons, indistinguishable from our Tao-RNAi results. In contrast, Rac1 overactivation strongly altered and reduced dendrite branching and also synapse density. Activation of the Ras pathway and loss of Tao have similar effects on the dendritic field and synapse numbers of A08n neurons without perturbation of overall synaptic density suggesting that Tao kinase likely controls concerted dendrite growth and synaptogenesis. In contrast, Rac1 overactivation did impair synaptogenesis more severely than dendrite growth, possibly due to cytoskeletal perturbations preventing synapse formation or stabilization. We have added the new data in Supplementary Figure 3. This argues that Tao and Ras are involved in dendritic growth but at least are also permissive for synaptogenesis as well. Of note however, we do think that Tao does perturb synaptic function and maybe also structure (as shown in mammals, see Yadav et al., Neuron 2016, Richter et al., Mol. Psych. 2018): in our case, loss of Tao resulted in mostly unchanged or slightly elevated numbers of C4da-A08n neuron synapses, yet significantly reduced physiological responses of A08n neurons (see new Supplementary Fig. 6B-D). In addition, expressing kinase-impaired TaoK2 (A135P) in a Tao-RNAi background did indeed uncouple dendritic overgrowth from synaptogenesis as indicated by the non-rescue of dendrite growth defects but normalization of A08n postsynaptic numbers (see Fig. 5C-

E, Supplementary Fig. 5). Therefore, we do think that besides dendritic growth, Tao function also affects some aspects of synaptogenesis/synaptic function.

3) It's unclear throughout (to non-aficionados) what else C4da connects to – could the authors make a discussion of this more prominent to understand the circuit a bit better?
Response: We agree with the reviewer and have included a subset of the C4da neuron circuit in our model (Fig. 7F) and added more information about the nociceptive circuit in our introduction/discussion (highlighted in yellow in the manuscript file).

4) Figure 4 (and graphs like it) are very hard to read. The authors should adjust the formatting to promote comprehension.
Response: We have increased the font size of the labels to increase clarity and readability of the graphs and figures throughout.

Reviewer #3 (Remarks to the Author):

In this study the authors interrogate molecular mechanisms that underlie synaptic scaling during development using a somatosensory circuit in Drosophila larvae. The authors first established light-microscopic methods for quantifying the synapses between larval nociceptors and one of its important second-order neurons, called A08n. They then described how synapse numbers change in larval development. They identify the kinase Tao as a negative regulator of A08n post-synaptic formation during developmental scaling, and demonstrate that the human homolog, but not its kinase-impaired autism-linked variant, rescues the defects caused by loss of Tao. They further show that loss of Tao activity results in circuit aberrations and behavioral changes.
These findings have significant implications for understanding the mechanisms of synaptic scaling during development. The results that show the functional conservation of mammalian and Drosophila Tao Kinase suggest the findings might be relevant to diseases. Most experiments are well designed (though see below), and the work is well articulated. The authors' savvy use of genetic approaches in interrogating synaptic and circuit functions is a strength of the work. I'd support the publication of this paper in Nature Communications after the authors address the following concerns.

Major concerns:

1) In several places the results from statistical analysis do not support the conclusions. The conclusions should be supported by statistical analysis, or they should be revised or removed.
p.7: "C4da neuron presynaptic puncta plateaued between 96 h and 120 h, while A08n postsynaptic counts dropped mildly at 120 h", but in Fig.2D there is no significant change between 96 h and 120 h.
Also, "C4da presynaptic output to A08n neurons remained steady across larval development", but in Fig.2E there are significant differences among 48 h, 72 h, 96 h and 120 h.
p.7: "In contrast, we observed a significant increase in synapse/postsynapse ratios for A08n neurons between 48 h and 96 h, indicating a gradual increase in…". However, the result indicates an increase from 48 h to 72 h, but did not compare between 48 hr and 96 hr.

p. 10: "density of Drep2-GFP-labeled A08n postsynapses suggesting that Tao activity did not strongly perturb synaptic density (Fig. S2D)". The result indicates significant change with one * in the figure.

Response: We thank the reviewer for pointing out the discrepancies in the text and the statistical analysis. We have carefully reanalyzed our data using appropriate statistical tests and adjusted our conclusions accordingly. Regarding the statement on p.10 on synaptic density, there is indeed no significant difference between all genotypes as we were referring to the number of postsynapses/μm^2, which are unchanged. However, we acknowledge that the figure labeling and wording might have been misleading and we improved both by adding the statistical significance (n.s.) in Supplementary Figure 2D and by changing the wording.

2) The authors used t-test throughout Fig.2, but ANOVA followed by Bartlett's post hoc should be used.

Response: We thank the reviewer for this comment. To our knowledge, Bartlett's test is used to check homogeneity of variances, which we have validated for the data in Fig. 2. We have now applied ANOVA with a Sidak's *post hoc* test in Fig. 2, as it allows comparison of selected pairs of means for appropriate comparison of the two genotypes at each individual time point.

3) To this reviewer, the correlation shown in Fig 1H & I are not strong enough to indicate a potential postsynaptic control of synaptic connectivity (p.6), and the stated implications of these results should be revised. The postsynaptic Drep2-GFP signals in A08n are much more punctate than the presynaptic Brp[short]-mCherry signals in C4da. It is thus possible that the quantification of Drep2-GFP was more accurate than Brp[short]. In addition, the method used for quantifying C4da-A08n synapses in these results, which is not described, may affect the result. If the synapses are counted by co-localization of Brp[short]-mCherry and Drep2-GFP, the presynaptic correlation might be weakened if C4da neurons have more post-synaptic partners than A08n have presynaptic partners.

Response: We mostly agree with the reviewer and have therefore tuned down our conclusions in this respect. We still feel the correlation is interesting and might be meaningful and have thus included the panels in Supplementary Figure 1D,E. The data were taken from the same synaptic marker analysis as shown in Fig.1 and were plotted to test for correlation between the number of detected pre- or postsynaptic spots with the number of colocalized C4da-A08n spots per hemisegment (within the synaptic threshold of 350nm). We have also updated our methods section to clarify that the correlation was derived from the same colocalization method used throughout our work. While we do not know the precise number of A08n presynaptic partners, C4da neurons have at least 13 different partner neurons (Gerhard et al. Elife 2016), of which A08n are a major subset (20-30% of all C4da synapses at L3 based on our analysis, see Fig. 1D). While we agree that the presynaptic Brp signal is less punctate, our numbers based on light microscopic colocalization analysis are nonetheless in good agreement with the ultrastructural EM data (see Fig. 1H).

4) The authors concluded that ectopic synapses between C3da and A08n are functional and contribute to nociceptive behaviors. However, the first two bars in Fig.7D show that activation of C3da in larvae with Tao-defective A08n neurons does not increase nociceptive behaviors, including rolling and bending, while it increases the turning associated with C3da, which is opposite to authors' conclusion.

Moreover, the first two bars in Fig.7E shows the opposite to the claim that defects in A08n

caused by Tao RNAi turns C4da-specific behaviors (nociceptive) towards C3da-specific behaviors (mechanosensory). In control, C3da-specific behaviors dominate nociceptive behaviors while Tao RNAi in A08n neurons increases the rolling behaviors (i.e., towards C4da-specific behaviors).

Response: We agree that our optogenetic behavioral data is non-intuitive and difficult to interpret. The primary effect of activating C3da/cho neurons is a full body contraction behavior, described as a noxious cold response (Turner et al. 2016, 2018). C3da dependent mechanosensory turning behavior is associated with local thoracic segment-specific activation with innocuous touch stimuli (Yan et al., Nature 2014). Optogenetic activation of all C3da/cho neurons mostly gives rise to full body contraction behavior as previously described. This behavior overrules C4da neuron-dependent rolling and normally negates any effect of C4da neuron co-activation. However, optogenetic stimulation of C3da/cho as well as additional activation of C4da also results in "mixed" behavioral responses (C3da dependent contraction/turning + C4da rolling/bending), likely due to common downstream outputs that are strongly activated by the optogenetic stimulation. While this type of activation is not very physiological, we hypothesized that additional inhibitory partner neurons in this circuit are affected non-autonomously by Tao manipulation in A08n, which might explain a C3da specific "gain of function" effect. We thus attempted to describe this in the associated model, but we are grateful to the reviewer for pointing out the logical flaws of our model.

Taking into account the comments of reviewer #1 and the concerns of reviewer #3, we therefore decided to remove these data from the revised manuscript (original Fig. 7D-F). We instead focused our study on "native stimuli" using mechanonociception and noxious cold stimulation, which both gave reliable and interpretable results. As outlined above (see response to reviewer #1), loss of Tao-induced C3da-A08n connectivity increased A08n neuron calcium responses to noxious cold and induced rolling behavior normally not observed in C3da/cho dependent cold responses. Together these data suggest that aberrant C3da-A08 neuron connectivity alters functional connectivity and behavioral responses to specific modalities (harsh touch vs. noxious cold). These data are included in the revised Fig.7C-E and Supplementary Fig.7E. However, we do not think that C3da neurons are the only ectopic partners of A08n under Tao-RNAi conditions, suggesting additional circuit changes might occur due to our manipulation. Thus we cannot rule out higher order network effects, which might contribute to these and additional behavioral changes.

5) There doesn't seem to be any basis for the inhibition loop from A08n to C3da/Ch projections in the model (Fig.7F). Although this inhibition loop explains why Tao RNAi in A08n increases rolling when C3da and C4da are activated together (Fig.7E), it does not explain why Tao RNAi in A08n decreases the rolling when C3da is activated alone (Fig.7D).

Response: We thank the reviewer for this comment and agree with his/her assessment. The model was mostly meant to interpret and explain the results stemming from the observed induction of C4da neuron-specific rolling during C3/4/cho co-activation and A08n Tao manipulation.

In light of the novel data presented in the revised manuscript and the insightful comments from all reviewers we have included an updated model (including additional known cho/C3da/C4da partners), which is consistent with our functional and behavioral findings (see revised Fig. 7E). While we currently cannot explain all behavioral changes, we think that the shift in modality-specific behavior is primarily due to ectopic C3da-A08n neuron synapses, which is reflected in the increase of noxious cold induced rolling behavior upon loss of Tao in A08n.

6) In p. 15 and Fig 6 (p. 17), NompC-lexA was used as a C3da driver. However, this driver expresses transgenes in both C3da and chordotonal neurons. The main text and the table for "Drosophila melanogaster stocks" should be revised to reflect this fact. Also, "NompC-lexAop" in the label of Fig 6A should be "NompC-lexA".
Response: We thank the reviewer for pointing this out and we have corrected the mistake.

Minor concerns:
1) p.5: The labels for Fig 1G-J are rather nondescript. For example, consider changing the y-axis labels for Fig 1H and I from "synapse" to "C4da-A08n synapses" to be clear about the type of synapses that were quantified. Make the same change in the other figures with the same issue (e.g., Fig 3).
Response: We have updated and improved the labeling of these figures accordingly.

2) Indicate the developmental stage of the larvae used in the experiments shown in Fig.3.
Response: We have now included the stage of the animals in Fig. 3 and other figure legends (96h AEL).

3) p. 27: "pUAttB" should probably be "pUASTattB".
Response: We have corrected the mistake.

4) p. 27: "For activity-dependent GFP reconstitution across synaptic partners…", but this reviewer didn't find the main text or figure legends mention activity-dependent GRASP.
Response: The Syb-GRASP method we used has been shown to be activity-dependent (MacPherson et al., Nat. Comm. 2015). However, using the published protocol we depolarized all neurons with high KCl buffer to get complete GFP reconstitution for synapse counting. Thus in our case the methods statement was just referring to the fact that Syb-GRASP labeling is activity-dependent.

5) It's not easy to tell what Fig 4G is intended to show. Can the authors label or explain in the legends the anterior-posterior orientation of the images?
Response: All hemisegments shown in our figures are anterior up and lateral to the left. In Fig. 4G', we projected the shown images in 4G along the Y axis to illustrate the ectopic localization of Drep2-GFP puncta under Tao-RNAi conditions in the XZ dimension (indicated by arrowheads in Fig. 4G and G'). For clarification, we added an "anterior" and "posterior" and "medial"/"lateral" labels in Fig.1 and Fig. 4G' to indicate the orientation of the shown images.

6) p.20, bottom 4 lines: "About half of all animals displayed mixed responses composed of successive combinations of bending followed by contraction and rolling. 51% of these larvae sequentially performed all three behaviors, with two thirds contracting before rolling." To this reviewer, one would expect about 25% larvae exhibiting rolling, based on this description. However, the % rolling shown in Fig 7D are much less than this number. Is the description on p.20 correct?
Response: The descriptions were indeed correct as they referred to only the fraction of animals displaying mixed behaviors. However, as discussed above we removed these data from our revised manuscript to clarify our results.

7) The development of C4da topography was first reported in Yang et al., (2014) Current Biology, 24(9): 1024-1030. That paper describes the presence of topography in early 2nd and early 3rd instar larvae (Fig S1F), which roughly match the 48 and 96 h AEL in this manuscript, and should be cited in discussions about the maintenance of topography during development, for example, in p. 22.
Response: We have added the citation to the relevant part of the discussion as suggested.

8) p.23: "the larval nociceptive network seems to rely more on functional rather than structural adjustments when challenged by sensory input alterations, although fine-scale topography of C4da neuron axon projections has been shown to require activity-dependent Trim9 function." Neither this study nor the Kaneko et al. cited in this sentence provides any evidence that supports the lack of activity-dependent adjustment by sensory input alternation. Moreover, the regulation of fine-scale topography of C4da neuron axon projections could be through spontaneous activities rather than sensory inputs.
Response: We apologize if our statement was misleading. In fact, we tried to argue that there is indeed evidence for activity-dependent adjustments in the nociceptive network. We have rephrased our discussion in this respect to clarify our point.

9) p.7: Typo- "…provide valid representations of C4da-A08n neuron connectivity"
Response: We have corrected the mistake.

10) p.15: Typo- "…first detectable at 48h AEL. ."
Response: We have corrected the mistake.

11) Figure 6B: The white circles are difficult to distinguish from the black squares. The groups should be marked more clearly.
Response: We have now used more discernible labels for the data points.

12) p. 21: "…contraction responses virtually indistinguishable from activation of C3da neurons alone…"
Response: This statement is no longer part of our manuscript as it referred to the deleted figure panels.

REVIEWERS' COMMENTS:

Reviewer #1 (Remarks to the Author):

Notes on Soba paper

The paper is much improved by the revisions, and could be published in its present form. I just have a few comments:

1) Despite the additional samples, the effect on Ca++ levels produced by Tao RNAi is still not very impressive (Supp. Fig. 6D). But I suppose it is simply a small effect. They did analyze a lot of preps.

2) They might want to move the last graph of Supp. Fig. 7, showing rolling, into the main figure. This is a better way to convey the data than Fig. 7D. It clearly shows that rolling is a novel behavior observed only with Tao RNAi. Fig. 7E makes the same point, but perhaps in a less obvious way.

3) I have to disagree with the other reviewer about Brp-short. The Brp-short pattern is very fuzzy, and resembles what is seen with full-length Brp overexpression. It looks less punctate than when endogenous Brp is labeled (for example with the STaR method). I think that, while Brp-short is a useful tool, it doesn't provide clear localization of presynapses. Drep2-GFP is a much cleaner marker for postsynaptic sites than Brp-short is for presynapses. They might want to use expansion microscopy, which works very well on larval brains, to get a clearer picture of the relationships between the Brp-short labeling and the Drep2 puncta.

Reviewer #2 (Remarks to the Author):

The authors have done a commendable job of addressing mine and the other reviewers' comments. I would recommend publication, though I would ask the authors to consider the one comment below - not a dealbreaker, but I would advocate for this.

The new experiments regarding Tao, Rac, and Ras are quite interesting. I think these experiments have a place in the literature, but it should be made more explicit that there may be no connection at all between Ras and Tao. They phenocopy, which is interesting, but may be otherwise unrelated. I think saying that Tao perturbation causes a Ras-like phenotype is too subtle and the language should be strengthened a bit more so that the reader does not artificially conflate Tao and Ras.

Reviewer #3 (Remarks to the Author):

The revised manuscript has addressed all my concerns. I support its publication in Nature Communications. The authors are to be congratulated for this important contribution to the field. (Bing Ye)

Point by point response

We would like thank the reviewers for their helpful comments. Please find below our point by point response.

Reviewer #1 (Remarks to the Author):

The paper is much improved by the revisions, and could be published in its present form. I just have a few comments:

1) Despite the additional samples, the effect on Ca++ levels produced by Tao RNAi is still not very impressive (Supp. Fig. 6D). But I suppose it is simply a small effect. They did analyze a lot of preps.

Response: We agree with the reviewer that the effect on calcium level responses of A08n neurons by Tao RNAi is small, yet we found consistent behavioral defects using either optogenetic activation of C4da neurons or mechanonociceptive stimulation (see Fig. 7A, Suppl. Fig. 7A,B) . We note that while synapse numbers between C4da and A08n neurons are fairly unchanged or mildly increased, their function is somewhat impaired by loss of Tao.

2) They might want to move the last graph of Supp. Fig. 7, showing rolling, into the main figure. This is a better way to convey the data than Fig. 7D. It clearly shows that rolling is a novel behavior observed only with Tao RNAi. Fig. 7E makes the same point, but perhaps in a less obvious way.

Response: The graphs in Supp. Fig. 7 show the onset of the observed behaviors depending on the temperature. As our main finding is that we observed pure rolling behavior with A08n-driven Tao RNAi, we do not think that these graphs properly represent it either. We now instead provide cumulative plots for C3da/cho-dependent contraction and C4da neuron-dependent rolling behavior for each genotype to better illustrate our point (new Fig. 7E). Former Fig. 7E is now in Supplementary Figure 7F.

3) I have to disagree with the other reviewer about Brp-short. The Brp-short pattern is very fuzzy, and resembles what is seen with full-length Brp overexpression. It looks less punctate than when endogenous Brp is labeled (for example with the STaR method). I think that, while Brp-short is a useful tool, it doesn't provide clear localization of presynapses. Drep2-GFP is a much cleaner marker for postsynaptic sites than Brp-short is for presynapses. They might want to use expansion microscopy, which works very well on larval brains, to get a clearer picture of the relationships between the Brp-short labeling and the Drep2 puncta.

Response: We agree that the Brp-short signal is less punctate than drep2-GFP. However, we respectfully note that the Brp-short label has been used in multiple studies and shows discrete localization to presynaptic active zones in different neurons (e.g. Christiansen et al., 2011; Hu et al., 2017; Kremer et al., 2010). Brp-short cannot localize to the active zone by itself, but requires binding to endogenous full length Brp. While we have not compared the Brp-short label with the STaR method in our system, we think the apparent "fuzziness" is rather an intrinsic property of the C4da sensory neuron active zones, which often stretch out due to the polyadic organization of C4da neuron synapses (see Suppl. Fig. 1F for EM images with the Brp-short compared to drep2 label and also Gerhard et al., 2017). We are not aware of a study comparing Brp-short and the STaR method in our system, however both have been used to label adult photoreceptor terminals and to us, the labeling results are highly similar (Sugie et al., 2015). Nonetheless, we acknowledge that depending on the levels of Brp-short, overloading the neurons due to strong overexpression might cause a higher background compared to endogenous labeling. Furthermore, we fully agree with the reviewer that expansion microscopy will certainly improve the resolution of Brp puncta and we very much appreciate the reviewer's suggestion for our future studies.

Reviewer #2 (Remarks to the Author):
The authors have done a commendable job of addressing mine and the other reviewers' comments. I would recommend publication, though I would ask the authors to consider the one comment below - not a dealbreaker, but I would advocate for this.
The new experiments regarding Tao, Rac, and Ras are quite interesting. I think these experiments have a place in the literature, but it should be made more explicit that there may be no connection at all between Ras and Tao. They phenocopy, which is interesting, but may be otherwise unrelated. I think saying that Tao perturbation causes a Ras-like phenotype is too subtle and the language should be strengthened a bit more so that the reader does not artificially conflate Tao and Ras.
Response: We thank the reviewer for this comment and we have now clearly stated in the results and discussion that a potential functional or genetic link between Ras and Tao requires further investigation.

Reviewer #3 (Remarks to the Author):
The revised manuscript has addressed all my concerns. I support its publication in Nature Communications. The authors are to be congratulated for this important contribution to the field. (Bing Ye)
Response: We very much thank the reviewer for his previous helpful suggestions to improve our manuscript.

References:

Christiansen, F., Zube, C., Andlauer, T.F.M., Wichmann, C., Fouquet, W., Owald, D., Mertel, S., Leiss, F., Tavosanis, G., Luna, A.J.F., et al. (2011). Presynapses in Kenyon cell dendrites in the mushroom body calyx of Drosophila. J. Neurosci. *31*, 9696–9707.

Gerhard, S., Andrade, I., Fetter, R.D., Cardona, A., and Schneider-Mizell, C.M. (2017). Conserved neural circuit structure across Drosophila larval development revealed by comparative connectomics. Elife *6*, 1–17.

Hu, C., Petersen, M., Hoyer, N., Spitzweck, B., Tenedini, F., Wang, D., Gruschka, A., Burchardt, L.S., Szpotowicz, E., Schweizer, M., et al. (2017). Sensory integration and neuromodulatory feedback facilitate Drosophila mechanonociceptive behavior. Nat. Neurosci. *20*, 1085–1095.

Kremer, M.C., Christiansen, F., Leiss, F., Paehler, M., Knapek, S., Andlauer, T.F.M., Förstner, F., Kloppenburg, P., Sigrist, S.J., and Tavosanis, G. (2010). Structural long-term changes at mushroom body input synapses. Curr. Biol. *20*, 1938–1944.

Sugie, A., Hakeda-Suzuki, S., Suzuki, E., Silies, M., Shimozono, M., Möhl, C., Suzuki, T., and Tavosanis, G. (2015). Molecular Remodeling of the Presynaptic Active Zone of Drosophila Photoreceptors via Activity-Dependent Feedback. Neuron *86*, 711–725.
